# Study of Structure, Physico-Mechanical Properties and Biocompatibility of Modified Cellulose-Based Conduits to Replace Injured Blood Vessels

**DOI:** 10.3390/polym18111389

**Published:** 2026-06-03

**Authors:** Marina V. Parchaykina, Mikhail A. Baykov, Elvira S. Revina, Maria V. Vedunova, Tatiana A. Mishchenko, Alena A. Sausheva, Parvina Z. Ashurova, Elizaveta I. Isaeva, Kirill D. Sinitsyn, Mikhail V. Shchankin, Natalia B. Nazarova, Alena O. Bogatyreva, Viktor V. Revin

**Affiliations:** 1 Department of Biotechnology and Biochemistry, Ogarev Mordovia State University, Saransk 430005, Russia; mikhail.baikov.a@yandex.ru (M.A.B.); rewina.elvira.s@yandex.ru (E.S.R.); sauschevaa@yandex.ru (A.A.S.); a.parvina@list.ru (P.Z.A.); isaeva_liza_2003@mail.ru (E.I.I.); spartanez64@yandex.ru (K.D.S.); schankinmv@mail.ru (M.V.S.); n.sapunowa2016@yandex.ru (N.B.N.); bogatyrevaao@mail.ru (A.O.B.); revinvv2010@yandex.ru (V.V.R.); 2 Institute of Biology and Biomedicine, Lobachevsky State University of Nizhny Novgorod, Nizhny Novgorod 603022, Russia; mvedunova@yandex.ru (M.V.V.); saharnova87@mail.ru (T.A.M.)

**Keywords:** bacterial cellulose, conduits, injured blood vessels, SEM, oxygen permeability, biomedicine, biocompatibility

## Abstract

The article is devoted to the study of the structure, physico-mechanical properties and biocompatibility of modified conduits based on bacterial cellulose (BC) to replace injured blood vessels. It has been shown that both samples have almost the same elastic recoil and are superior to synthetic vascular grafts in terms of the parameters studied. It should be noted that the first modified sample is characterized by greater elasticity and lower tensile strength compared to the second sample; however, the physico-mechanical properties of the obtained conduits are in the range corresponding to native blood vessels. Scanning electron microscopy (SEM) demonstrated that the conduits under study had a fibrillar structure with nanosized pores that enabled the adhesion of endothelial cells on the internal surface of the vascular implant, improved elasticity under transverse pressure, and raised the elasticity modulus when stretching along the fibrils. Thermogravimetry revealed that elastic recoil formation depended on the nature of polyvinyl alcohol (PVA) interaction with the nanofibrillar structure of BC rather than on the content of polyvinyl alcohol used for modification. The MTT test results confirmed no cytotoxicity and high oxygen permeability in the studied samples, opening great opportunities for their application in regenerative biomedicine to replace injured blood vessels.

## 1. Introduction

Most solutions in modern biomedicine aim to regenerate injured tissues and organs [1,2,3,4]. At present, the urgent issue is the production and investigation of natural and synthetic conduits serving as scaffolds for targeted regeneration and used to replace injured tissue and organ regions [5,6]. Currently, one of the crucial problems worldwide is the prevalence of cardiovascular and cerebrovascular diseases, as well as the atherosclerosis of peripheral vessels [7,8]. One solution to the problem is to introduce into the body artificial structures that have the properties needed to replace native blood vessels [9,10].

An ideal blood vessel analog should have high mechanical strength, expansibility, the ability to recover its original shape (elastic recoil), elasticity, correspondence with autologous blood vessels, thermal stability, biocompatibility, particularly cytocompatibility and hemocompatibility for the rapid proliferation of endothelial cells, and oxygen permeability [11]. Traditionally, autologous blood vessels, particularly the saphenous vein and the radial artery, have been used; however, their application is limited by systemic diseases, the underlying cause of injured blood vessels [12,13].

Synthetic polymer materials remain the basis for producing vascular conduits used in medicine. Despite their widespread application, none of these materials can completely replicate the biomechanical and physiological properties of native vessels. Moreover, they appear to be more susceptible to infection in surgeries [14].

As rightly noted in modern literature, the creation of small-diameter functional vascular grafts remains one of the most difficult tasks in the field of regenerative medicine, and this problem has persisted for more than 70 years since the first attempts at the clinical use of synthetic prostheses. The main difficulty lies in the fact that while large diameter grafts (>6 mm) demonstrate acceptable long-term patency due to high blood flow, which reduces the risk of thrombosis, small-diameter grafts face fundamentally different hemodynamic conditions [15]. The primary cause is an enlargement of the tunica intima of the vessels due to the migration and proliferation of smooth muscle cells, which in turn reduces vascular patency. Other causes include biomechanical factors, such as wall stress and shear stress on the vessel walls. The simplest way to solve this problem is to match the mechanical parameters as closely as possible to those of the vessels [9].

Polytetrafluoroethylene is one of the most widely used materials for vascular prostheses due to its high medical strength, resistance to degradation and durability. However, its hydrophobic properties and insufficient elasticity prevent full endothelization, raising the risk of thrombosis and neointimal hyperplasia [16]. Polyethylene terephthalate is a polyether that has been used in vascular surgery for decades due to its high strength and stability, although its low biocompatibility and elasticity significantly limit its application in practice [17].

Biomaterials are natural polymers widely used in tissue engineering due to their similarity with the extracellular matrix. Additionally, a considerable part of them can act as conductors for some time before being gradually replaced by native tissues. The most widely investigated materials are collagen, elastin, fibrin, fibroin, polylactic acid and bacterial cellulose [18,19,20].

Collagen exhibits high biocompatibility and hydrophilic properties, and it has specific binding sites for the effective adhesion of endothelial cells. However, its mechanical strength is insufficient for use under high hemodynamic loads, especially in the arterial system. The application of elastin or its derivatives in vascular conduits enables approximation of their mechanical properties to those of native vessels; nevertheless, its application is limited by the complexity of preparation and storage. Fibrin matrices exhibit high biocompatibility and contribute to active cell migration and proliferation; however, their low mechanical strength and rapid degradation restrict their application [21]. Polylactic acid is an aliphatic polyether with good biocompatibility and degradability to nontoxic products, although it has high hydrophobic properties that hamper vascular prosthesis endothelization [22]. Polycaprolactone is a semicrystalline aliphatic polyether characterized by slow degradation, although its hydrophobic properties and low cellular activity prevent effective tissue remodeling [23].

One of the most promising biomaterials is BC, a polysaccharide with a 3D nanofibrous structure that exhibits high purity and crystallinity compared with traditional plant cellulose. Due to its biocompatibility, chemical stability and inactivity, viscoelasticity and hydrophilic properties, BC can be used for wound healing and the replacement of sclerotic blood vessels, which appear to be extremely urgent in the context of the everyday world [24,25]. BC properties are appropriate for use in the body as a blood vessel analog due to the structural peculiarities of the polysaccharide under study. BC is characterized by a nanofibrous structure with a hydrophilic surface and sufficient porosity, providing a proper space scaffold and mechanical support for cell growth and adhesion, as well as high permeability to gases and liquids, one of the key parameters of the conduits used. The polysaccharide crystallinity provides durability and biostability to the patient’s enzyme systems [26]. Moreover, due to hydroxyl groups, BC can undergo various modifications, in particular, using PVA, citric acid and glycerin. PVA is a synthetic, bioinert, and hydrophilic polymer approved by the FDA for biomedical applications. Unlike collagen, which is often characterized by low mechanical strength and polylactide, which is hydrophobic and creates an acidic environment during degradation, PVA does not cause inflammation and maintains pH stability under physiological conditions. Moreover, the presence of numerous hydroxyl groups in the PVA chain makes it an ideal base for chemical modification [27]. Citric acid is selected as a stapler. Unlike toxic glutaraldehyde or carbodiimides, citric acid is a natural metabolite of the Krebs cycle and is approved by the FDA as a safe substance [28]. During heat treatment, its carboxyl groups enter into an esterification reaction with the hydroxyl groups of PVA, forming strong covalent bonds. This transforms the soluble PVA into an insoluble three-dimensional hydrogel capable of maintaining a given mechanical shape. Glycerin, in turn, acts as a bioinert plasticizer. Its molecules are embedded between the PVA chains, weakening the hydrogen bonds and increasing the mobility of the polymer segments. This increases the elasticity and flexibility of the material, which is critically important for realizing the reversible shape-memory effect. This approach has been widely tested in the literature for the creation of wound coatings and shape-memory hydrogels, which confirms its validity and prospects for biomedical applications [29].

Thus, the purpose of the present investigation was to study the structure, physico-mechanical properties and biocompatibility of modified conduits based on BC to assess its ability to withstand physiological loads, maintain integrity during implantation, and provide the necessary elastic recoil and immunogenicity, which is one of the clinically significant requirements for vascular prostheses.

## 2. Materials and Methods

### 2.1. BC-Based Conduit Production

In the present study, *Komagataeibacter sucrofermentans* B-11267 was used in the BC-based conduit production. The *K. sucrofermentans* B-11267 strain was isolated from Kombucha and deposited in the Russian National Collection of Industrial Microorganisms (VKPM) (registration number: B-11267). The strain was cultured in media containing 50 g/L beet molasses at pH 4.5. The beet molasses used in the present study were obtained from the Romodanovsky Sugar Factory (Mordovia, Russia). The beet molasses had the following composition (% by weight): dry matter—82.0; sucrose—50.0; total nitrogen—2.3. Prior to inoculation, the media were autoclaved at 121 °C for 20 min. The inoculum for the seeding nutrient media was prepared using a method previously described [19]. BC-based conduits were produced by fermenting *K. sucrofermentans* B-11267 in a container with two 200 cm silicone tubes with an internal diameter of 5 mm, using a molasses medium with a concentration of 50 g/L. The medium was inoculated with 10% (*v*/*v*) inoculum. For this purpose, 1350 mL of the liquid nutrient medium and 150 mL of the inoculum were added to the container under aseptic conditions. Each silicone tube contained 39 mL of the culture medium. *K. sucrofermentans* B-11267 was cultured for 7 days with double mixing of the nutrient medium in each tube for 10 min on days 1 and 2. For mixing, we used a peristaltic dosing pump LOIP LS-301 (LOIP, St. Petersburg, Russia) at a rotor speed of 200 rpm. At the end of the process, the nutrient medium and the resulting gel film were removed from the container, and 1500 mL of distilled water was added. To remove the silicone tubes from the tubular structures, we carried out a process similar to mixing the medium, pushing the biopolymer out of the tubes with a stream of water. The resulting structures were processed to remove the nutrient medium cells and components using a method previously described [20]. The obtained BC-based conduits were modified in several ways. The first modifying solution was prepared by mixing water, 50 mL; PVA (up to an end concentration of 2%); citric acid, 0.2 g. and glycerin, 3.0 g. To prepare the second solution, 15–20 mL of water and 2.25 g of dry PVA were heated with stirring, then 0.75 g of citric acid was added, and the solution was stirred for 5–10 min until large agglomerates were dissolved. The temperature was brought up to 40–50 °C, followed by the addition of pure glycerin, 0.95 mL. Between experiments, the obtained samples were kept in a refrigerator at 4 °C.

Then, to fix a certain mechanical shape of the samples, the following procedure was performed. The first sample was kept in the corresponding modifying solution for 30 min and then dried at 120 °C for 30 min. The second sample was kept in the corresponding solution for 1.5 h and then dried for 30 min at 115 °C.

**Figure 1:**
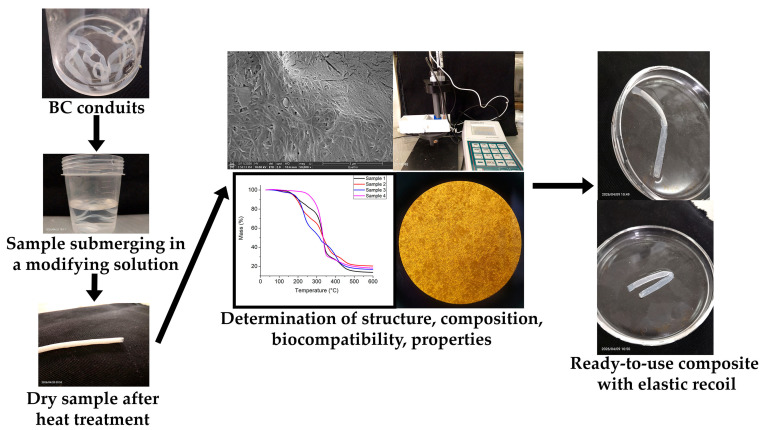
Scheme of modification and investigation of modified cellulose-based conduits to replace injured blood vessels.

Furthermore, the obtained composites were fixed onto Teflon tubes with a smaller diameter than the samples. We used glass as an alternative; however, it made it difficult to remove sample from the glass without damaging it. Moreover, PETG rods were considered, although plastic melting resulted in an improper constant shape. To facilitate removing the samples from the tubes, they were soaked in distilled water. The obtained BC tubular structures were autoclaved at 121 °C for 20 min (Figure 1).

### 2.2. Strength and Tensile Measurement

To measure strength, the samples were cut into 2 × 1 cm rectangles. The average thickness was measured using a high-resolution automatic thickness gauge, the CHY-C2 Thickness Tester (Labthink, Jinan, China), by recording the values for each sample in several places.

After cutting, the BC-based conduits were assessed on a PARAM^®^ XLW (PC) Auto Tensile Tester (Labthink, Jinan, China). The samples were fastened in the machine forceps and stretched at 50 mm × min^−1^ until they ruptured.

For soft hydrated materials, the use of relatively high tensile speeds is a common practice, as these materials exhibit pronounced viscoelastic properties. At low strain rates, significant stress relaxation can occur, leading to the underestimation of the mechanical properties. In the literature, test speeds ranging from 10 to 100 mm/min have been used for vascular grafts and bacterial cellulose, depending on the sample geometry and hydration conditions [9,30,31].

In particular, Oliveira Barud et al. used a speed of 50 mm/min for testing bacterial cellulose membranes for biomedical applications. Similar strain rates were also used in the mechanical characterization of vascular substitutes and compliant hydrogel materials. Additionally, the ISO 7198:2016 standard [32] (“Cardiovascular implants and extracorporeal systems—Vascular prostheses”) does not specify a single fixed test speed, but rather recommends selecting conditions based on the viscoelastic properties and geometry of the vascular material being studied [33].

We used the obtained data to calculate Young’s modulus to describe the material’s ability to resist deformation, compression and tensile forces, which is significant for conduits. The calculations were made using [34]

(1) $$E=\frac{Fl}{S\Delta l},$$

where F—force applied to rupture the sample, l—length, S—the sample square, and Δl—the sample tensile prior to the rupture.

### 2.3. Scanning Electron Microscopy

The microstructure of BC-based conduits was studied using a QuantaTM 3D 200i multifunctional raster electron microscope (Thermo Scientific, Waltham, MA, USA).

### 2.4. Thermogravimetric Analysis (TGA)

Thermogravimetric analysis was performed using a TG 209 F1 Libra thermobalance (Netzsch, Selb, Germany).

### 2.5. MTT Assay

To determine cytotoxicity, we used the NGUK-1 cell line, obtained from Gasser’s node neurinoma (schwannoma), representing a tumor culture of neuroectodermal origin, and characterized by high proliferative activity and pronounced oncogenic properties.

To determine the biocompatibility of the samples, they were placed in sterile water for 7 days, followed by bringing the sodium chloride (NaCl) concentration to a final concentration of 0.9%, corresponding to a saline solution.

At the end of the incubation, we performed a standard passage of cell culture followed by cell inoculation into a 96-well plate. The cells were equidistributed and then incubated under standard culture conditions (37 °C, 5% CO_2_) for 72 h to achieve the required adhesion and recovery after reinoculation.

After 3 days, the culture medium (supernatant) was carefully removed to minimize mechanical effects on the cellular monolayer and prevent damage or delamination.

A series of serial dilutions of the studied sample was then prepared in seven wells with a dilution coefficient of 10. To prepare the first dilution, we used a volume ratio of 10 µL of the studied substance to 90 µL of 0.9% NaCl solution, yielding an initial concentration corresponding to the computed dilution pattern.

DMEM (PanEco, Moscow, Russia), 100 µL, was placed in each well of the plate. An extra 150 µL of the studied extract at the initial concentration was added to the first column. The solutions, with concentrations zero times lower than those of the previous well, were added to the subsequent columns, forming a concentration gradient over the whole row. After adding the solution, the well contents were carefully mixed by pipetting to avoid air bubbles. The plate was incubated for 2 days.

After the exposure period and removing the culture medium, 10 µL of working solution (MTT, Sigma Aldrich, St. Louis, MO, USA) was added to each well. The solution volume and concentration were adjusted so that the end MTT concentration in each well was 0.45 mg/mL. The reagent was carefully added to avoid bubble formation and mechanical damage to the cell layer.

The plate was incubated at 37 °C for 1–4 h under standard culture conditions. During this period, mitochondrial dehydrogenase in metabolically active cells reduced MTT to insoluble violet formazan crystals, which were accumulated inside the cells.

After incubation, we added 100 µL of the solution to each well to solubilize (dimethylsulfoxide, DMSO) and dissolve the formazan crystals. After the formazan had completely dissolved, the optical density was spectrophotometrically measured at a wavelength of 570 nm (Multiskan FC Thermo Scientific, Waltham, MA, USA). The obtained values were used to quantitatively assess cell viability, with the findings expressed as percentages relative to the control group.

### 2.6. Oxygen Permeability Measurement

The oxygen permeability of the studied BC-based conduits was assessed on an oximeter DO210E with a sensor DO-957-Q (Fcombio, Zhengzhou, China) in saturation and concentration measurement modes. The operating principle of the device is that oxygen enters the electrolyte zone through the membrane, followed by reduction at the cathode.

The obtained BC-based conduits were cut into rectangles, 1 × 2 cm in size, the measuring surface of the device was 201.06 mm^3^ (taking into account the membrane diameter of 16 mm), for further assessment of the required characteristic, with accuracy up to 2% or 0.3 mg/L of the chosen equipment; partial pressure gradient was 0.28 atm/mm.

Each sample was tightly rested against the electrode membrane; after having made sure that the BC-based conduits were completely seated to the measuring section of the device, we recorded the value 15 min after starting the measurement. The thickness of each sample used in the measurements is shown in Table 1 and Table 2. All measurements were carried out at a temperature of 36.6 °C. Between the measuring procedures, the samples were kept in cold (up to 4 °C) distilled water to prevent them from drying and developing pathogenic microbial flora.

### 2.7. Statistical Analysis

All presented data are averages of at least three experimental runs, with 10 replicates per mean. Standard deviations of the mean were calculated using Microsoft Excel 2013 (Microsoft Corporation, Redmond, WA, USA). The obtained data were statistically analyzed using two-way ANOVA followed by Tukey’s multiple-comparison test for pairwise group comparisons, with a significance threshold of 5%.

## 3. Results and Discussion

The clinical significance of the problem of prosthetics of blood vessels is due to the annually growing need for vascular shunts for the treatment of coronary heart disease and peripheral vascular diseases against the background of limited availability of autologous options (subcutaneous vein, internal thoracic artery) in patients with advanced atherosclerosis, diabetes mellitus or previous operations. It should be emphasized that none of the currently available approaches, including commercially available synthetic grafts made of polytetrafluoroethylene and polyethylene terephthalate, as well as tissue engineering structures, is able to fully solve these problems, which makes the development of new biocompatible materials with programmable mechanical properties an extremely urgent task. BC has attracted considerable attention due to its high mechanical strength, excellent biocompatibility, ability to retain moisture, and nanofibrillar structure resembling an extracellular matrix [35]. These properties make BC especially attractive for creating scaffolds in vascular tissue engineering. According to literature data, composite grafts made of bacterial nanocellulose and polyurethane maintained patency for 9 months after implantation in rats, demonstrating rapid endothelialization and decreased expression of proinflammatory cytokines [7]. The first stage involved analyzing the structure and physico-mechanical properties of the obtained samples. These parameters are of great importance, since they determine the sample’s capability to endure physical exertion (particularly pulse pressure and tensile), preserve integrity in implantation and provide the necessary elastic recoil, i.e., the ability to recover its initial shape after stretching. It is impossible to predict the conduit response in blood flow and its resistance to expansion or rupture without such analysis. The summary data are presented in Table 1 and Table 2.

According to the data in these tables, the first sample had the highest tensile value (49.56 ± 5.24%) at a quite low Young’s modulus (1.69 ± 0.25 MPa). Thus, it was the most elastic, although least rigid, sample, which could be useful to imitate the plasticity of native vessels. The second sample was characterized by the highest Young’s modulus (2.70 ± 0.43 MPa) and tensile strength (17.13 ± 0.37 N) values at a relatively low tensile value (14.93 ± 0.68%). Thus, it was the strongest and most rigid that minimized the risk of aneurysmal dilation under perfusion pressure.

The morphology and structural organization of bacterial cellulose-based biocomposites were studied by SEM. Micro- and nanostructure assessment of the obtained samples is crucial for understanding their capacity for elastic recoil and predicting their operational suitability as vascular implants [36].

Materials used in vascular surgery must endure cyclic hemodynamic loads while simultaneously retaining shape after stretching, which is known to depend directly on the fibrillar frame features [37,38]. The three-dimensional mesh structure of BC with pore diameters in the nanometer range provides effective distribution of mechanical load along the fibrils, and interfibrillar links participate in restoring the preliminary set shape.

**Figure 2:**
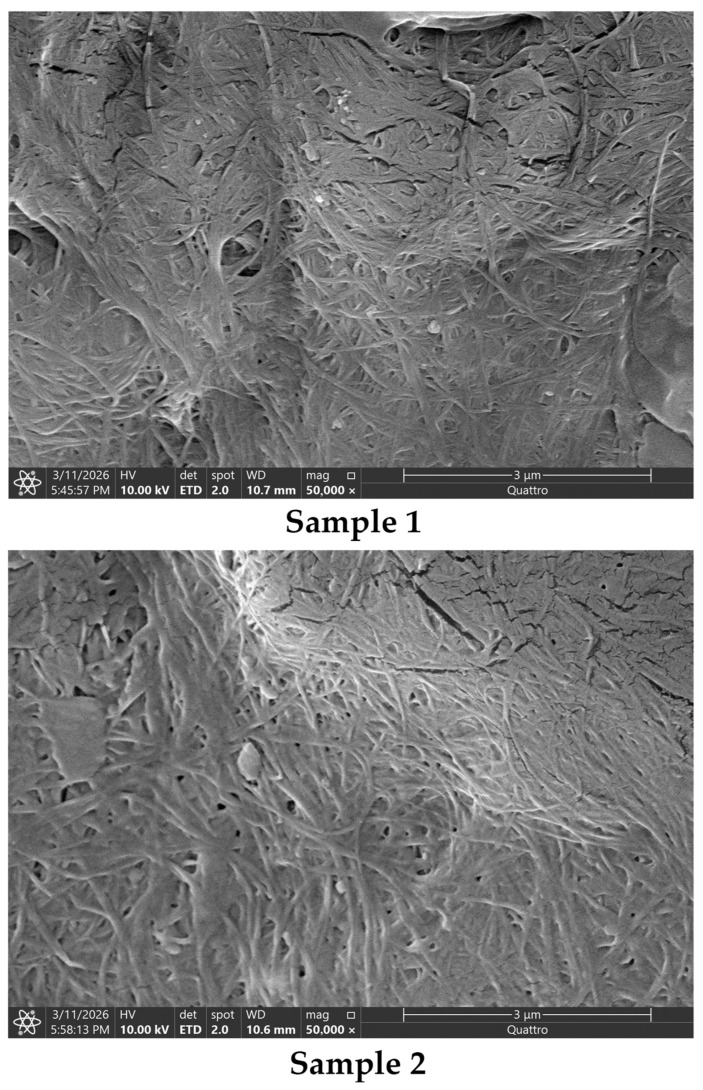
SEM image of modified BC-based conduits.

Scanning electron microscopy revealed that samples 1 and 2 have micro- and nanostructures typical of BC (Figure 2).

The average pore size is 100 nm for the first sample and 90 nm for the second sample; for both samples, the pore diameter was 64 nm. The porosity was 4% for the first sample and 29% for the second sample. Taking the density of the material (BC) as 1.6 g/cm^3^, we measured the scaffold density and calculated it using the following formula [39]:

(2) $$P=1-\left(\frac{\rho_{scaffold}}{\rho_{material}}\right)\;$$

**Figure 3:**
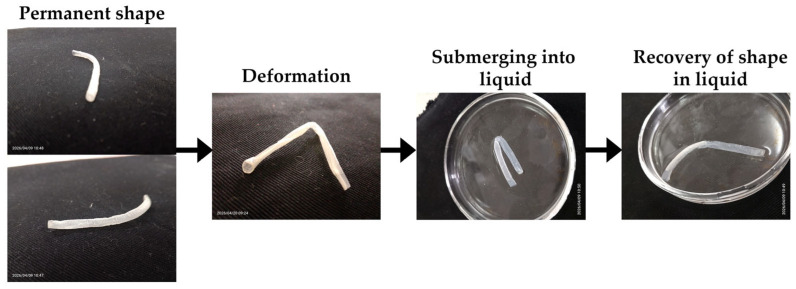
Modified cellulose-based conduits with elastic recoil.

The presence of a fibrillar architecture with nanosized pores created a high surface area, which is of great importance for endothelial cell adhesion during neointima formation on the internal surface of the vascular conduit. The fibers oriented in a certain way and the porosity assigned anisotropy to the mechanical properties: under transverse pressure, the elasticity increased, while stretching along the predominant fibril orientation increased Young’s modulus. It is effective for imitating the mechanics of native vessels. Thus, the necessary condition for elastic recoil in the obtained biocomposites is the preservation of the native three-dimensional nanofibrillar structure of the bacterial cellulose. It can be concluded that the obtained samples had the capacity for cyclic deformity under intravascular pressure and subsequent return to their initial position, which is of great significance for preventing thrombus formation and aneurysmal dilation (Figure 3).

The next experimental stage involved thermogravimetric analysis, enabling quantitative assessment of the thermal stability of the biocomposites and determination of the mass fraction of some components in the dry constituent of the material under study. This is of primary importance for vascular implants since the ratio of components directly affects the manifestation of mechanical and physiological properties. Moreover, the analysis enabled precise evaluation of material contamination with any chemical substances. According to the temperature intervals of the destruction of the main components of the modifying solutions, particularly 150–220 °C for citric acid, 210–290 °C for glycerin and 280–330 °C for PVA, we calculated the mass fractions of substances in the dried composites.

**Figure 4:**
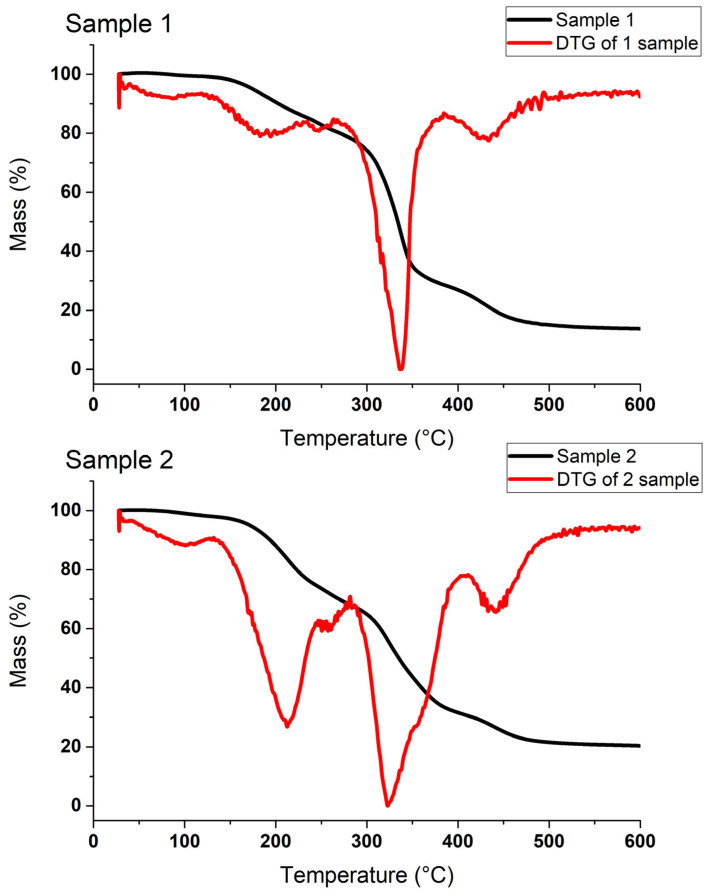
Thermogravimetric curves of modified BC-based conduits.

In the first sample, the PVA content was 51.3%, citric acid was 13.4% and glycerin was 5.3%. Thus, the total additive content (about 18.7%) provided high elasticity, making it appropriate for clinical situations when increased compliance of a vascular prosthesis is required. The second sample had the lowest PVA content (36.6%) compared with the first sample; however, it exhibited the best physico-mechanical parameters. This suggests that the key role in elastic recoil formation is played by PVA interaction with the nanofibrillar structure of bacterial cellulose, rather than the absolute PVA level. The plasticizer content in the sample (about 28.1%) provided sufficient elasticity while preserving the strength characteristics of the material (Figure 4).

Thus, the nature of intermolecular interactions in a biocomposite, rather than absolute concentrations of plasticizers, plays the key role in manifesting mechanical and biological properties. Hydrogen bonding is the most important for imparting elastic recoil to PVA, as it helps impart a fixed phase to hold the material’s permanent shape, whereas the PVA chains act as a reversible phase. The presence of PVA detected by thermography was the threshold at which our elastic recoil functioned [40].

We compared whether parameters such as permeability and pore diameter correlate, as well as whether the Young’s module and fibril diameter correlate. Spearman’s correlation shows that for the first sample, the initial oxygen permeability strongly correlated with the pore size at 0 min r = 0.70337, *p* = 0.02324, as well as at 5 min, r = 0.8471, *p* = 0.00198; at 15 and 30 min, no correlation was observed *p* > 0.05. For the second sample, no stable dependence was observed except for 15 min—r = 0.63298 at *p* = 0.04949. We also tested the correlation between the fibril size and diameter—but no correlation was observed for the first and second samples.

The Young’s modulus of the samples was closest to that of the Great saphenous vein (GSV). According to the literature, the tensile stress of the first sample was within the range of values for the GSV (~1–4 MPa) and internal mammary artery (IMA) (~2–12 MPa), human carotid artery (0.4–1.2), human coronary artery (0.3–1 MPa), and native small-diameter vessels (0.2–12 MPa). The first sample significantly exceeded all of the aforementioned veins in this parameter. The tensile force of the first sample was closer to the IMA and human carotid artery, while the second sample significantly exceeded native veins in this parameter. The elongation of the first sample was significantly close to the lower scapulae of the aforementioned vessels, while the second sample did not have the extensibility of native vascular tissues [41,42,43,44,45].

The structure of the vascular scaffold and its mechanical compliance directly impact hemodynamics, permeability, cellular infiltration, and vascular remodeling [44,46]. Sample 1 exhibits mechanical properties most similar to native vessels. Despite a relatively high tensile stress (7 MPa), GSV—2.4 MPa, IMA—4.1 MPa. The sample is characterized by 50% elongation (whereas 60–80 for GSV and 40–70% IMA) and a Young’s modulus of 1.69 MPa, which corresponds to the range of compliant vascular tissues. This combination of strength and elasticity potentially contributes to a more physiological transmission of pulse deformation and a reduction in compliance mismatch, which is considered a key factor in neointimal hyperplasia and impaired long-term patency of vascular grafts [46].

Sample 2, although possessing a high tensile force (17 N), is characterized by significantly lower extensibility (14%) and a higher Young’s modulus (3 MPa), indicating more rigid mechanical behavior. According to modern mechanophysical models, increased vascular conduit stiffness may be associated with less favorable microstructural organization and reduced permeability, potentially limiting oxygen diffusion, endothelialization, and tissue integration [47].

Compared with clinically used synthetic vascular grafts such as ePTFE and Dacron, both studied samples demonstrated significantly lower stiffness and mechanical behavior closer to native vascular tissue. Sample 1 demonstrated the most favorable biomimetic profile due to a combination of a relatively low Young’s modulus, high extensibility and sufficient tensile strength, which may indicate better compliance and compatibility with physiological vascular pulsatile deformation. According to the literature, excessive stiffness and compliance mismatch of synthetic grafts are closely associated with hemodynamic impairment, the development of neointimal hyperplasia, and a decrease in the long-term patency of vascular grafts. In contrast, the more compliant mechanical behavior of sample 1 could potentially provide more favorable conditions for vascular remodeling, oxygen transport, and endothelial integration. Despite the higher tensile strength of sample 2, its increased stiffness and low extensibility indicate a more rigid conduit architecture, which is potentially less favorable for physiological vascular mechanics and mass transfer processes [48,49,50].

The present study involved an in vitro MTT test to assess the cytotoxicity of the obtained samples. The test was based on the recovery of yellow tetrazolium salt (MTT) by dehydrogenases of living cells into purple formazan crystals, the quantity of which was determined photometrically at a certain wavelength. For vascular conduits, cytotoxicity assessment on cell lines, particularly NGUK-1, enables the evaluation of the general biocompatibility of a biocomposite, exclusion of the subsequent release of toxic chemical compounds, and estimation of the impact of some cells on metabolism [51].

The research carried out on cell culture NGUK-1 over a concentration range from 10^0^ to 10^−6^ found that none of the obtained samples showed significant toxicity in the full concentration range. Therefore, the optical density (OD) values of the controls were comparable to those of other dilutions, and no classical dose-dependent toxicity was observed, as exhibited by the absence of a gradual OD decrease when the concentration increased.

The first sample was characterized by a metabolic activity curve lying close to the control, with a marked peak at the concentration of 10^−1^. At maximum concentration, OD reached the control value (OD ≈ 0.0502), suggesting no cytotoxicity in the cell culture under study and a possible low stimulating effect.

The second sample had a metabolic activity curve lying slightly above the control, with marked peaks at concentrations of 10^−5^ and 10^−3^. At maximum concentration, optical density approached the control level, reinforcing the absence of toxicity and suggesting the presence of a stimulating effect on cell metabolism.

Thus, both samples exhibited no cytotoxic effect on the NGUK-1 cell culture across the whole concentration range and no inhibitory effect on cell metabolism. The cell survival rates of the first and second samples were 97.7% and 98.7%, respectively.

The present study also assessed the permeability of vascular conduits for gases, particularly oxygen. A native vessel wall is known to have selective permeability and provide gas exchange between blood and surrounding tissues [52,53]. Accordingly, high oxygen permeability of the implanted materials prevents hypoxia in the mural area, maintains the viability of endothelial cells and provides physiologically appropriate metabolic conditions for vascular wall cells. Additionally, this capability of the biocomposite indirectly characterizes its porosity and hydrophilic properties, both of which are important for performing all transport functions. Table 3 and Table 4 present the summary data.

All samples were characterized by sufficient oxygen permeability, with mean values narrowly ranging from 9.9 ± 0.5 to 11.4 ± 0.5 mg/L, which is within statistical accuracy. Permeability tended to decrease with time, from 11.4 ± 0.5 to 10.6 ± 0.5 mg/L for the first sample, and from 11.0 ± 0.5 to 9.9 ± 0.5 mg/L for the second sample. This decrease could be due to the equilibrium gas concentration achieved on either side of the membrane and to diffusion flow cessation.

According to the literature, the obtained values are within the normal range and can provide appropriate gas exchange between blood and perivascular tissues, as well as prevent hypoxia of the implanted conduit wall.

## 4. Conclusions

The present study examined the structure, physico-mechanical properties and biocompatibility of conduits modified by different substances to replace injured blood vessels. It was shown that the first modified sample has a relatively high elasticity and a lower Young’s modulus compared to the second sample, which is characterized by significantly lower elasticity and a high Young’s modulus, indicating its more rigid structure. Nevertheless, both samples are characterized by almost the same ability to return to their original shape, and the studied physical and mechanical parameters are in the range corresponding to native blood vessels, surpassing synthetic vascular prostheses. Scanning electron microscopy demonstrated the presence of a fibrillar architecture with nanosized pores, which is of great importance for endothelial cell adhesion during neointima formation on the internal surface of vascular conduits. The fibers oriented in a certain way, porosity improves elasticity under transverse pressure, and the elasticity modulus increases when stretched along the fibrils, correlating with the findings on the strength of the samples. Moreover, the samples were characterized by high oxygen permeability required for proper physiological processes and exhibited no cytotoxicity. Thus, modified BC-based conduits can be considered as a promising material for vascular surgery; however, further studies related to the assessment of hemolysis and platelet adhesion are needed to eliminate the risk of thrombosis. In the future, we plan to conduct cyclic tensile loading–unloading tests in order to quantify elastic recoil, as well as suture retention strength testing to confirm surgical applicability and structural integrity. For use in vascular tissue engineering, the resulting conduits must meet the requirements for parameters such as suture retention strength, cyclic fatigue, and compliance. A critical factor for the effective use of vascular grafts is their successful endothelialization, and therefore, it is necessary to conduct tests aimed at seeding the obtained conduits with endothelial cells and evaluating the physiological characteristics of vascular tissue. Despite the large amount of future research, the results described in this article confirm the possibility of using modified BC-based conduits as vascular prostheses, which seems to be a promising area of regenerative biomedicine.

## Figures and Tables

**Table 1:** The values of physico-mechanical parameters of the first modified BC composite sample.

No	Thickness, µm	Tensile, %	Tensile Pressure, MPa	Tensile Strength, N	Young’s Modulus, MPa
1	134.40	26.00	6.79	5.73	0.82
2	294.00	64.00	2.07	7.94	2.15
3	129.47	48.09	8.64	7.46	1.64
4	147.12	50.00	5.58	7.22	1.79
5	118.92	52.40	9.29	7.47	1.73
6	150.83	50.20	7.45	7.80	1.84
7	89.03	53.00	5.77	7.74	1.52
8	163.19	52.10	4.74	7.45	1.52
9	93.39	48.40	10.40	7.73	1.95
10	128.68	51.40	9.58	7.10	1.96
M ± m	144.90 ± 35.11	49.56 ± 5.24	7.03 ± 2.04	7.36 ± 0.41	1.69 ± 0.25

**Table 2:** The values of physico-mechanical parameters of the second modified BC composite sample.

No	Thickness, µm	Tensile, %	Tensile Pressure, MPa	Tensile Strength, N	Young’s Modulus, MPa
1	110.00	12.50	3.00	18.00	2.73
2	100.00	15.30	2.50	16.0	4.27
3	210.00	16.10	3.20	17.40	1.39
4	139.20	14.60	3.00	17.40	2.87
5	163.30	15.50	3.10	16.98	2.67
6	167.90	14.70	2.90	16.93	2.58
7	167.90	15.40	3.10	17.45	2.38
8	136.30	15.60	3.00	17.14	2.56
9	142.30	15.10	2.90	16.73	2.44
10	140.90	14.50	2.90	17.26	3.08
M ± m	147.78 ± 23.60	14.93 ± 0.68	2.96 ± 0.13	17.13 ± 0.37	2.70 ± 0.43

**Table 3:** Oxygen permeability values of the first modified BC composite sample (mg/L).

No	0 min	5 min	15 min	30 min
1	12.6	11.6	11.4	11.2
2	11.6	11.4	11.1	10.9
3	9.2	8.8	8.5	8.2
4	11.2	10.7	10.5	10.9
5	11.7	11.5	11.0	11.0
6	12.1	11.4	10.3	10.5
7	11.7	11.7	10.8	11.0
8	11.3	11.7	10.2	10.5
9	11.2	11.0	10.4	10.5
10	11.4	10.9	10.4	11.2
M ± m	11.4 ± 0.5	11.1 ± 0.6	10.5 ± 0.5	10.6 ± 0.5

**Table 4:** Oxygen permeability values of the second modified BC composite sample (mg/L).

No	0 min	5 min	15 min	30 min
1	11.6	10.8	10.4	10.2
2	11.5	11.1	10.8	10.5
3	9.2	8.8	8.5	8.2
4	11.6	10.3	10.3	9.7
5	10.7	10.6	10.2	10.1
6	11.3	10.9	10.1	10.0
7	11.2	10.8	10.3	10.4
8	11.3	10.3	10.1	9.7
9	10.8	10.6	9.9	9.7
10	10.9	11.0	9.9	10.4
M ± m	11.0 ± 0.5	10.5 ± 0.4	10.0 ± 0.4	9.9 ± 0.5

## Data Availability

Sequence data are available from GenBank, NCBI.
